# The new SUMPOT to predict postoperative complications using an Artificial Neural Network

**DOI:** 10.1038/s41598-021-01913-z

**Published:** 2021-11-22

**Authors:** Cosimo Chelazzi, Gianluca Villa, Andrea Manno, Viola Ranfagni, Eleonora Gemmi, Stefano Romagnoli

**Affiliations:** 1 Department of Anesthesia and Intensive Care, Azienda Ospedaliero Universitaria Careggi, Florence, Italy; 2 Department of Health Sciences, Section of Anesthesiology, Intensive Care and Pain Medicine, University of Florence, Florence, Italy; 3 Center of Excellence Dews, Department of Information Engineering, Computer Science and Mathematics, University of L’Aquila, L’Aquila, Italy

**Keywords:** Health care, Risk factors

## Abstract

An accurate assessment of preoperative risk may improve use of hospital resources and reduce morbidity and mortality in high-risk surgical patients. This study aims at implementing an automated surgical risk calculator based on Artificial Neural Network technology to identify patients at risk for postoperative complications. We developed the new SUMPOT based on risk factors previously used in other scoring systems and tested it in a cohort of 560 surgical patients undergoing elective or emergency procedures and subsequently admitted to intensive care units, high-dependency units or standard wards. The whole dataset was divided into a training set, to train the predictive model, and a testing set, to assess generalization performance. The effectiveness of the Artificial Neural Network is a measure of the accuracy in detecting those patients who will develop postoperative complications. A total of 560 surgical patients entered the analysis. Among them, 77 patients (13.7%) suffered from one or more postoperative complications (PoCs), while 483 patients (86.3%) did not. The trained Artificial Neural Network returned an average classification accuracy of 90% in the testing set. Specifically, classification accuracy was 90.2% in the control group (46 patients out of 51 were correctly classified) and 88.9% in the PoC group (8 patients out of 9 were correctly classified). The Artificial Neural Network showed good performance in predicting presence/absence of postoperative complications, suggesting its potential value for perioperative management of surgical patients. Further clinical studies are required to confirm its applicability in routine clinical practice.

## Introduction

Appropriate perioperative planning of elective post-operative admission to intensive care units (ICUs), high-dependency units (HDUs) or standard wards after non-cardiac surgery may improve postoperative outcomes in patients at risk for postoperative complications (PoCs)^1–5^. A clear body of evidence shows that elective postoperative ICU admission reduces the incidence of PoCs, while delayed or emergency admission to ICU/HDU following surgery may lead to worse outcomes^6^.

There is therefore a critical need for strategies to improve preoperative identification of those patients who are at high risk for PoCs through a more thoughtful use of available ICU/HDU resources^7^. Traditionally, the prediction of postoperative risk for complications and the identification of high-risk patients have been largely empirical and based on medical judgement, particularly for elective, non-cardiothoracic patients. Even though the clinical judgement of the attending physician retains great importance for assessing the perioperative risk of individual patients, this may be not specific and certainly does not allow for the standardized allocation of available ICU/HDU beds^8^. Several scores are available for risk stratification and identification of patients at risk for PoCs. Among them, the American Society of Anesthesiology-physical status (ASA-ps) and the Physiological and Operative Severity for the Enumeration of Mortality and Morbidity (POSSUM) have a long history of clinical use; however, they do show drawbacks and limitations^9,10^. Briefly, the ASA-ps is consistent with the clinical judgement on the global health status of the patient, irrespective of surgical procedure. The POSSUM score is more detailed and takes both patient- and surgery-related factors into account; however, it tends to overestimate mortality^9^. The more recent American College of Surgeon-Veterans Affairs National Surgical Quality Improvement Program surgical risk calculator (ACS-NSQIP) appears to be the most reliable system currently available, but the input of data may be cumbersome. It requires a precise preoperative definition of the ongoing surgery and may miss complications that do not fall into specific and pre-defined areas^11,12^. Furthermore, the tool has not been validated for use outside the United States.

In 2015, an easy to apply score, the Anesthesiological and Surgical Post-Operative Risk Assessment (ASPRA), has been implemented to evaluate the risk of PoCs based on type of surgery and comorbidity^13^. The main advantage of this tool was its easy and immediate applicability for risk stratification, taking into account both surgery- and patient-related risk factors. Statistical validation of the score was prospectively run within a validation set of 1928 surgical patients and showed a high positive predictive value to predict the occurrence of postoperative complications. An ASPRA score > 7 predicted the occurrence of PoCs in > 84.3% of cases. Moreover, Spearman’s correlation test performed on patients in the validation set showed a strong correlation between higher ASPRA scores and severity of PoCs, as defined by the Clavien-Dindo classification^13^.

For use in practice, a prognostic tool should be easy-to-use and directly applicable at the bedside. With these concepts in mind, we explored the potential of using new technologies (such as machine learning) to develop a new tool for risk assessment, named SUMPOT (SUrgical and Medical POstoperative complications prediction Tool). Based on Artificial Neural Network (ANN) technology, SUMPOT is aimed at supporting physicians in perioperative care planning through automatic assessment of risk factors for PoCs in patients undergoing surgery. The advantages of a neural network are threefold: automation; ability to reproduce the complex and hidden nonlinear relationship between risk factors and PoC events; and self-learning capability for progressively more accurate assessment of PoCs.

The aim of the present study was to assess the efficacy of SUMPOT in preoperatively identifying those patients who are at risk for postoperative complications. To this aim, the SUMPOT performance is assessed by comparing it with a Binary Decision Tree (BDT) predictive model. This comparison is motivated by the fact that ANNs and BDTs represent two opposite learning paradigms: the former have very strong predictive power, while the latter are much more interpretable.

The remainder of the paper is organized as follows. The methodology adopted in this study is contextualized and described in “Methods” section. In “Assessment of the SUMPOT” section we first describe the data collection and elaboration processes together with the experimental settings implemented to asses the SUMPOT performance, then we report the SUMPOT results compared with the binary decision tree method. The final “Discussion and conclusions” section is devoted to discussions and conclusions.

## Methods

### Background

Machine Learning (ML) is the study of computer programs that learn from experience^14^. Over the last few decades, ML techniques have been applied to many fields, including healthcare, energy, and transportation^15–18^.

As an example, we can consider a system which produces a certain output in correspondence to an input, according to an unknown functional relationship. Assuming that a dataset of historical input-output pairs, namely the training set, is available, ML techniques use the training set to generate (i.e. train) a surrogate model of the function, i.e. a model that approximates the behavior of the system. This process is called training phase. The model is then used to predict the unknown output for any different input combinations. Since the actual output associated to every sample of the training set is known, this type of ML techniques is denoted as supervised learning.

The prediction performance of the trained model, also called generalization, is measured on the testing set, i.e. a set of samples not used to train the model, for which both input and output are known.

The training phase is a challenging task, one that is commonly formulated as a mathematical optimization problem. One of the main difficulties during this phase concerns the overfitting phenomenon. When overfitting occurs, excessive effort is dedicated to the training phase, meaning that the resulting model is extremely accurate in reproducing the training data but is poor in terms of generalization. Other issues may involve lack or imbalance of data, or lack of an actual functional relationship between input and output.

ML is frequently used for classification. In classification, all samples belong to different classes, so that the output of each sample assumes a value in a finite set of categorical elements respresenting the different classes . A proper prediction model trained for classification should be able to reconstruct the unknown class membership (output) for any given sample (input). The most commonly used ML techniques for classifications are Decision Trees^19^, Support Vector Machines (SVMs)^20–22^, ANNs^23–25^, and recently Deep Neural Networks (DNNs)^26^. Even if all the above methods have been often applied to classification task in healthcare domains^27–30^, the Neural Networks based ones seem to be the more suited to capture the complicated and hidden nonlinear relationship between the input and the output, and are therefore the most used in this kind of applications. DNNs, which are mainly used for complicated image recognition and time series forecast applications^31,32^, have the strongest computational and representative power, but their training phase can be computational expensive and prone to numerical issues^33,34^ that may compromise the quality of the predictive model. In this study, as we sought for simpler models, easy-to-use for not ML practitioners and sufficiently accurate at the same time, we drove out choice to ANNs. In particular we have applied a Single Layer Feedforward Network (SLFN) with the training algorithm proposed by Grippo et al.^24^ and denoted as DEC(2). The usage of a SLFN is strengthened by the adoption of DEC(2) since, as will be better clarified below, DEC(2) is able to reduce the potential limitations of such model and to build good quality predictive models.

In the investigated case study we deal with binary classification, i.e. the samples belong to two different classes, conventionally denoted with labels 0 and 1.

### Overview on ANNs and on the adopted training algorithm

ANNs are characterized by a learning mechanism inspired by biological neurons. Briefly, ANNs are generally structured as networks of interconnected formal neurons, in which the formal neurons are processing units organized into ordered layers, while the connections between neurons are weighted (the signal flowing through the connection is multiplied by a coefficient called weight) and oriented (the flow has a specific direction). The connections are oriented from the first layer (input layer) to the last layer (output layer). Hence, an ANN works as an input-output system that receives input signals and produces an output. The output is the result of the propagation of the input signals from the input layer to the output layer. During this forward propagation, each neuron processes the weighted sum of all the signals coming from its ingoing connections by means of a mathematical function (activation function), and then produces an output signal that is transmitted to the neurons of the subsequent layer. The problem of training an ANN lies in tuning the weights of the connections between neurons so as to minimize the so called loss function, which measures the overall discrepancy between the outputs produced by the network in correspondence of the training inputs and their corresponding actual outputs.

SLFNs are the simplest ANN architectures as they are composed of only the input layer, a single hidden layer and the output layer. Although it is well known that SLFNs possess the universal approximation property^35^, i.e. they can approximate any continuous function with arbitrary precision, their training is not trivial. Indeed, as it is the case for general ANNs, minimizing their loss function is very difficult as the latter is highly nonconvex, with steep-sided valleys and flat regions, so that a training algorithm is likely to get stuck in poor quality solutions. Moreover, as mentioned before, overfitting phenomena may occur. In addition, it is worth pointing out that the SLFNs training algorithms are generally based on a procedure called backpropagation^23^ (used to iteratively reconstruct the gradient of the loss function) which is very time consuming.

As it is shown in^36^, training algorithm DEC(2), by adopting an intense decomposition approach and proper regularization techniques, is able to cope with all the previous drawbacks, allowing to quickly obtain good quality models. In particular, the decomposition has a strong impact on the computational time and aids the algorithm to escape from the attraction basin of poor solutions, while the regularization allows to generate simpler and more general models preventing overfitting.

## Assessment of the SUMPOT

### Data collection and preprocessing

This retrospective, observational, single center study was approved by the local ethics committee of Careggi University Hospital (CEAVC Largo Brambilla 3, Florence. Protocol number 2017-4010; date of approval 21/11/2017). All participants in the study returned a signed informed consent at the time of preoperative evaluation, prior to data collection. We retrospectively collected data from the medical charts of all consecutive in- and outpatients aged >18 who underwent elective, emergency, general or urologic surgical procedures from July 1 to October 1, 2017, and who were subsequently admitted to intensive care units (ICUs), high-dependency units (HDUs) or standard wards at the tertiary care teaching hospital of Careggi (Azienda Ospedaliero-Universitaria di Careggi), Florence, Italy.

For each enrolled patient, presence/absence of each of the risk factors already explored in the literature and mainly identified through the ASPRA score^13^ was assessed by scrutinizing the anesthetic record charts filled during preoperative examination (Table 1). This information formed the input data set of the SLFN. We considered only the presence or absence of any of the risk factors on a binary basis (i.e. 0 for absence of the risk factor, 1 for presence of the risk factor).

According to most recent literature, some modifications were done on the original risk factors previously identified in the ASPRA score. Total protein serum concentration $<7$ g/dL and perioperative weight loss $>10\%$ were considered as input risk factors to better estimate the nutritional status and frailty of the patient^37^. Age and sex were not included among the risk factors. Unstable coronary syndromes were excluded from patient-related input factors since they usually require treatment prior to elective surgery.

Due to the high number of minimally invasive robot-assisted surgical procedures performed in our center, we introduced the surgical approach to the procedure as a new binary input parameter for postoperative risk-assessment. Data were collected from open, laparoscopic, robotic, and endoscopic surgeries with details on the specific procedure. Each surgical procedure received a score of 1 or 0, depending on whether they were used or not. Data concerning the type of surgical technique were extracted from the preoperative plan and do not necessarily correspond to those actually applied.

The few continuous variables included among the risk factors were dichotomized so as to better fit the remaining binary factors, thus avoiding numerical issues during the mathematical operation involved in the training phase. Body Mass Index (BMI, calculated as usual) was dichotomized as normal or altered if $<17$ or $>25$ respectively. Elevated serum creatinine value was considered a binary input risk factor if greater than 1.5 md/dL.

According to this modification the new input data set of the SFLN was composed from 41 items, listed in Table 1.

Nine of the 41 input data were removed from the dataset, since no patients underwent the corresponding surgical procedure thus adding no information (in particular 19, 20, 22, 24, 25, 29, 31, 34, 35).

**Table 1:** The considered risk factors.

**Patients comorbidity factors**	
1. Abnormal ECG (left bundle branch block, left ventricular hypertrophy, repolarization abnormalities, non-sinus rhythm)	
2. Untreated hypertension or hypertension not controlled by medical therapy	
3. Previous thromboembolism	
4. Stable or controlled angina	
5. Previous myocardial infarction with no clinical or diagnostic evidence of residual ischemia	
6. Compensated heart failure or previous heart failure	
7. Diabetes mellitus	
8. Neoplastic disease	
9. Transfusion in the preoperative period (> 4 units)	
10. Smoking and/or drug addiction	
11. BMI > 25 or < 17 weight loss>10% in the preoperative period, plasma proteins < 7 g/dl	
12. Creatinine > 3.5 g/dl	
13. Steroid use	
14. Prevision of prolonged surgery (reoperation, anatomical abnormalities, etc.)	
15. History of COPD/dyspnea	
16. Decompensated heart failure	
17. Valve disease	
18. Severe arrhythmias: advanced AV block (second-degree block, Mobitz 2 > 2:1, block grade III)	
Symptomatic ventricular arrhythmias, supraventricular arrhythmias with uncontrolled ventricular response	
**Surgical procedures factors**	
19. Breast surgery	
20. Dental surgery	
21. Endocrine surgery (no pheochromocytoma)	
22. Eye surgery	
23. Gynecological surgery	
24. Reconstructive surgery	
25. Minor orthopedic surgery (other than hip and spine)	
26. Minor urological surgery	
27. Abdominal surgery	
28. Carotid surgery	
29. Head and neck surgery	
30. Neurologic/orthopedic (hip and spine) surgery	
31. Lung/kidney transplant	
32. Major urological surgery	
33. Endocrine surgery (pheochromocytoma)	
34. Peripheral vascular surgery	
35. Aortic and major vascular surgery	
36. Pancreas/liver surgery	
37. Emergency abdominal surgery	
**Surgical technique factors**	
38. Open surgery	
39. Laparoscopic surgery	
40. Robotic surgery	
41. Endoscopic surgery	

Each patient was monitored during the postoperative period until hospital discharge so as to evaluate any potential postoperative complications.

PoCs were classified according to the Clavien-Dindo classification of surgical complications that assigns a Clavien grade to each patient (Table 2). The entire cohort was divided into two classes corresponding to a Clavien score of $\le$ 1 (the control group) and > 1 (the PoCs group). In so doing, we addressed the binary classification problem (Table 2).

**Table 2:** Distribution of risk factors in the control group and the Pocs group.

Risk factors	Total	Control group (Clavien-Dindo $\le$1)	Pocs group (Clavien-Dindo > 1)
Abnormal ECG	188	168 (89.4%)	20 (10.6%)
Previous thromboembolism	17	16 (94.1%)	1 (5.1%)
Untreated hypertension or hypertension therapy	247	227(91.9%)	20 (8.1%)
Previous myocardial infarction without residual ischemia	26	26 (100%)	0 (0%)
Stable or controlled angina	21	19 (90.5%)	2(9.5%)
Compensated heart failure or previous heart failure	21	20 (95.2%)	1(4.5%)
Diabetes mellitus	64	57 (89%)	7(11%)
Neoplasticdisease	308	277(89.9%)	31(10.1%)
Transfusion in pre-operative period (> 4 units)	9	7 (77.7%)	2(22.3%)
Previous transient ischemic attack (TIA)/stroke	22	21 (95.5%)	1(4.5%)
Smoking addiction and/or drug addiction	180	165 (91.6%)	15 (8.4%)
BMI > 25/< 18, weight loss > 10%, plasma proteins < 7 g/dl	277	255 (92%)	22 (8%)
Creatinine > 1.5 mg/dl	45	41 (91%)	4 (9%)
Steroid use	29	25 (86%)	4 (14%)
Prevision of prolonged surgery	12	8 (66.6%)	4 (33.4%)
History of COPD/dyspnea	99	89 (89.9%)	10 (10.1%)
Decompensated heart failure	2	1 (50%)	1 (50%)
Symptomatic valvular disease	0	0	0
Severearrhythmias:	0	0	0
Endocrine surgery (no pheochromocytoma)	1	1 (100%)	0 (0%)
Gynecological surgery	4	3 (75%)	1 (25%)
Minor urological surgery	158	153 (96.8%)	5 (3.2%)
Abdominal surgery	249	223 (89.5%)	26 (10.5%)
Thoracic surgery	1	1(100%)	0 (0%)
Head and neck surgery	11	11(100%)	0 (0%)
Lung/kidney transplant	1	1 (100%)	0 (0%)
Mayor urological surgery	74	68(91.9%)	6 (8.1%)
Pheochromocytoma	1	1 (100%)	0 (0%)
Pancreas/liversurgery	24	19 (79.2%)	5 (20.8%)
Emergency abdominal surgery	2	0 (0%)	2(100%)
Open surgery	142	125 (88%)	17(22%)
laparoscopic surgery	143	132 (92.3%)	11(7.7%)
Robotic surgery	89	81 (91%)	8(9%)
Endoscopicsurgery	152	146 (96.7%)	6(3.3%)

For layout reasons, some risk factors descriptions have been abbreviated with respect to Table 1.

Data from a total of 526 surgical patients were entered into the study database. Based on the Clavien-Dindo score, 43 out of 526 patients (8.2%) suffered from one or more PoCs, while 483 patients (91.8%) did not. The final database was composed of 331 males and 194 females; mean age was 63 years. Table 2 reports the distribution of risk factors in the control group and the PoCs group. Table 3 summarizes the complications experienced by the PoC group, while Table 4 reports the distribution of complications among patients according to the Clavien-Dindo. classification.

**Table 3:** Clavien scores.

Grade	Definition
Grade I	Any deviation from the normal postoperative course without the need for pharmacological treatment, or surgical, endoscopic, or radiological procedures. Permitted therapeutic regimens: drugs and antiemetics, antipyretics, analgesics, diuretics, electrolytes, and physiotherapy. This grade also includes wound infections opened at the bedside
Grade II	Requiring pharmacological treatment with drugs other than those permitted for grade I complications
Grade III	Requiring surgical, endoscopic, or radiological procedures. Grade IIIa: procedure not under general anesthesia Grade IIIb: procedure under general anesthesia
Grade IV	Life-threatening complications (including central nervous system complications) requiring IC/ICU management. Grade IVa: single-organ dysfunction (including dialysis) Grade IVb: multiorgan dysfunction
Grade V	Death

**Table 4:** Distribution of complications among patients, according to the Clavien-Dindo classification.

Clavien score	Number of cases
0–1	483 (91.8%)
2	25 (4.7%)
3	12 (2.3%)
4	3 (0.6%)
5	3 (0.6%)

The whole dataset has been divided into a training set (to train the predictive model) and a testing set (to assess generalization performance, see “Methods” section). The training set consisted of 466 patients, of whom 34 suffered from one or more PoCs (i.e. Clavien > 1, 7.3%) and 432 did not (i.e. control patients, 92.7%). The testing set consisted of 60 patients, of whom 9 suffered from one or more PoCs (15%) and 51 (85%) did not. The samples of the testing set have been selected randomly in both classes. In order to overcome data imbalance in the training set, data from the PoC group were duplicated according to an oversampling strategy (27). As a result, the final dataset was composed by 561 patients. Of these, 60 patients entered the testing set and 500 patients, of whom 68 (i.e. 34 times 2) belonged to the PoC group (see Table 5) and 432 to the control group, entered the training set. This artifice could be introduced on the grounds that it does not impair the generalization accuracy of the input-output mathematical relation found by the trained SLFN. On the contrary, if the classes of the training set are imbalanced, the minimization process may tend to favor the minimization of errors associated to the largest class with respect to the less represented one, thus producing a model which is accurate for one class and not for the other. Oversampling the less represented class allows to allocate more weight to its errors so as to mathematically force the training phase toward a more balanced model^38^.

The distribution of patients in the final training and training sets is provided in Table 6.

**Table 5:** Distribution of the Clavien-Dindo score among patients, according to the ASA score.

ASA score	Clavien-Dindo score	
	$\le$ 1	> 1
1	66 (94.3%)	4 (5.7%)
2	294 (93.3%)	921 (6.7%)
3	118 (87.4%)	17 (12.6%)
4	5 (83.3%)	1 (16.7%)
5	0	0

**Table 6:** Distribution of patients in the training set and the testing set.

	Training set	Testing set
Patients from the control group	432	51
Patients from the PoCs group	68	9
Total	500	60

### Experimental settings

A grid-search fivefold cross-validation technique^14^ has been used to determine the best hyperparameters of the SLFN. In particular, a finite set of candidate values for each hyperparameter is initially specified by the user. For each possible combination of values of hyperparameters among the considered candidate sets (the points of the ideal grid), a fivefold cross-validation procedure is used to assess the quality of the model for such combination. In the fivefold cross-validation, the training set is partitioned into 5 partitions, and 5 different new training sets (trials) are obtained by removing from the original training set one partition at a time. At each of the 5 cross-validation steps, the ANN is trained on one of the trials and validated with a performance score on the corresponding removed partition (validation set). The final cross-validation score is the average of the scores obtained on the 5 different validations sets. The combination of values obtaining the best cross-validation score is selected, and then the ANN model is trained over all the original training set and tested on the separate testing set.

As in^36^, the neurons of the hidden layer of the SLFN have been equipped with a sigmoidal activation function of the form

$$\begin{aligned} \frac{1}{1+e^{-\beta x}} \end{aligned}$$

where $\beta$ is a given parameter. The values of the hyperparameters explored in the grid-search was

- $\{15, 35, 55, 75\}$ as the number of hidden layer neurons,
- $\{1, 2, 3, 4\}$ as $\beta$ coefficient in the activation function,
- $\{0.0, 0.1, 0.01, 0.001\}$ as the weighting coefficient of the regularization terms,
- $\{10, 20, 30, 40\}$ as the maximum number of macro-iterations of the training algorithm.

The better configuration determined by the grid-search cross-validation procedure was 35 hidden layer neurons, $\beta =3$, 0.01 as the regularization coefficient, and 20 macro-iterations. It is worth mentioning that the natural output of ANNs is continuous, however, a binary classification is easily obtained by adding a filter in which outputs greater than a certain threshold are assigned to one class and outputs less than the threshold are assigned to the other class. In this study, since samples were assigned either class 0 or class 1, we used a threshold of 0.5 for the filter.

### Performance measures and comparisons

The accuracy of the system in predicting the development of postoperative complications is expressed as accuracy rate, i.e. the rate of patients correctly classified by the SLFN. Specifically, “positive accuracy” (also denoted as sensitivity) was defined as the rate of correct preoperative predictions of complications in patients who did actually experience PoCs while “negative accuracy” (also denoted as specificity) was defined as the rate of correct preoperative predictions of absence of complications in patients who experienced an uneventful postsurgical course (i.e., control cases). Besides positive and negative accuracies, we consider also mean accuracy, balanced accuracy, positive predicted value (PPV), negative predicted value (NPV), ROC and AUC criteria.

Since the SLFN model has strong representation power with scarce interpretability, in order to better assess and contextualize its performance in relation to the investigated case study, we compared the SLFN performance to that of BDTs^39^.Without entering into details, BDTs are structured as a sequence of binary decisions operated in correspondence of nodes interconnected according to a hierarchical tree-based structure. At each decision node there is a bifucraction of the tree, indeed the node has 2 outgoing connections. The decision mechanism is such that each decision node is associated to a predictor variable, and a sample passing through that node is forwarded to its left connection if the corresponding predictor variable assumes a value higher than a certain threshold (or assumes value 1 if the variable is binary), otherwise (or if it assumes value 0 in the binary case) it is forwarded to the right connection. Starting from the first decision node (root node), each sample goes through a path in the tree according to the decision mechanism of the nodes. The paths along the tree end in different terminal nodes (leaf nodes) associated to one of the 2 classes, so that each sample will be associated to the class of the terminal node in which it falls. Training a BDT essentially consists in determining the predictor variable associated to each decision node and the corresponding threshold (if the variable is not binary), and like ANNs is performed in a supervised learning manner.

As already mentioned, the rationale behind this comparison is that the BDT approach is the opposite to the ANN one. Indeed, BDTs are characterized by a high level of interpretability, but they generally lack accuracy; this is essentially due to the mathematical complexity of the BDTs training optimization problem and to the absence of nonlinear input-output mappings in their structure that makes model architecture too simple. The purpose of this comparison was to evaluate the extent to which the adoption of ANNs can increase accuracy of the predictive model at the expenses of interpretability.

The DEC(2) training algorithm used in the experiments is the FORTRAN custom implementation directly obtained from its authors, while the BDT was implemented with the function FITCTREE of the MATLAB Statistics and Machine Learning Toolbox.

Lastly, we collected the ASA scores of all enrolled patients (Table 5 reports the distribution of PoCs in patients according to the ASA score), as reported in the anesthetic record chart, and compared them to the ANN model for prediction of postoperative complications.

### Results

The SLFN equipped with DEC(2) training algorithm obtained an average classification accuracy of 90% in the testing set (54 patients out of 60 were correctly classified), 90.2% in the control group (46 patients out of 51 were correctly classified), and 88.9% in the PoC group (8 patients out of 9 were correctly classified). The trained BDT model obtained an average classification accuracy of 83.3% in the testing set (50 patients out of 60 were correctly classified), 94.1% in the control group (48 patients out of 51 were correctly classified), and 22.2% in the PoC group (2 patients out of 9 were correctly classified).

**Figure 1:**
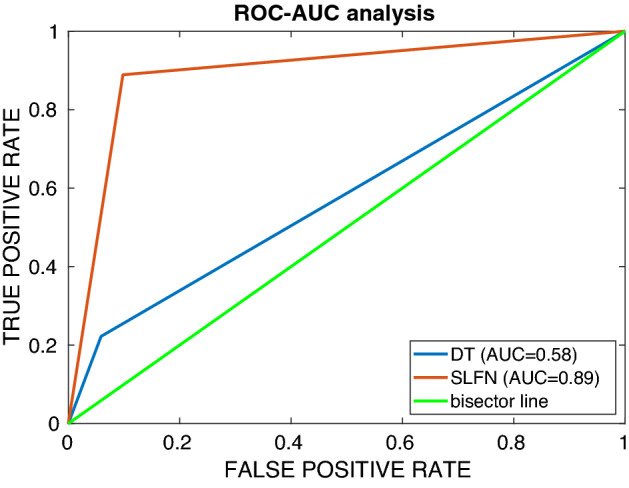
ROC-AUC analysis for the SLFN and DT models.

The following confusion matrices highlight the performance of both methods with respect to different criteria. 
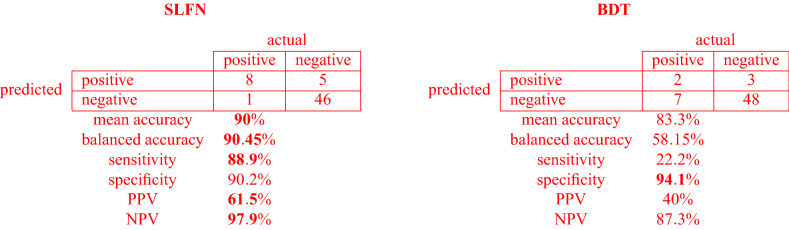
 Figure 1 shows a much higher ROC curve for the SLFN than for the BDT, corresponding to a much large value of the AUC (0.89 and 0.58 respectively).

### Ethics approval

The study was performed in accordance with the principles of the Declaration of Helsinki and was approved by the local ethics committee (Comitato Etico di Area Vasta Centro, Largo Brambilla 3 Florence). Protocol number: 2017-4010. Date of approval: 21/11/2017).

### Consent to participate

Informed consent was obtained from all participants if subjects are under grade 4th and 5th, from a parent and/or legal guardian.

## Discussion and conclusions

This study aimed at implementing an automated surgical risk calculator based on ANN technology to define patients’ individual risk for postoperative complications.

High-risk patients undergoing non-cardiac surgery represent a large share of admissions to ICUs in more developed countries^40^. A thorough preoperative assessment followed by an adequate monitoring strategy are paramount to improve perioperative management of surgical patients, improve postoperative outcomes and reduce morbidity, mortality, and related health costs^41^. Postoperative complications may be linked to patient-related factors and surgical-related factors. These factors can be detected at the time of preoperative assessment, provided a clear surgical plan has been made^42^. Another crucial factor is the intraoperative course of surgery; however, preoperative analysis of risk factors does not consider unpredictable intraoperative events (e.g. bleeding from accidental vascular injury)^43^. Since most complications originate in the first 48 hours, with hypoxia/hypotension occurring most frequently, a planned ICU/HDU admission may be a beneficial option to allow prompt detection and timely management of the ongoing issue^3^. Accurate identification of patients at high risk for PoCs remains difficult. Recent papers have emphasized that less than 15% of “high-risk” surgical patients are electively admitted to the highest level of postoperative care, but account for 80% of perioperative deaths^44^. Although elective postoperative ICU admission may be standard practice for some subsets of high-risk patients (e.g. after cardio-thoracic or emergency surgery), for other patients this is not the case. In the setting of non-cardiac surgery, clinical scores may represent valuable clinical tools to identify those patients who could benefit from more intensive levels of postoperative care.

Over the last few years, several large international trials have been planned or are underway to define the best approach to preoperative assessment. Recently, Kahan et al.^45^ reported the results of a secondary analysis of the International Surgical Outcome Study (ISOS) database, in which the authors aimed to shed light on the relationship between critical care admission and perioperative mortality in a large cohort of elective surgical patients. Interestingly, no direct association was found between the two variables. Although presenting some limitations, the study by Taccone et al.^46^ made it clear that routine postoperative admission to ICUs is not helpful “per se”. The authors underline the need for tailored postoperative admission based on the risk factor of each individual patient, and thus stress the importance of accurate postoperative risk prediction^47^.

There are several scores that can be used to predict a complicated postoperative course. In general, these scores correlate the probability of PoCs to the general health status of the patient, and/or to the expected complexity of the planned surgery. The most widely used scores include the ASA-ps classification, the original and modified versions of the POSSUM score, and the APACHE score for patients already admitted to postoperative ICUs^48–50^. Despite their widespread use, however, these scores show certain limitations, including a high inconsistency between ratings^48,49^, and the tendency to under- or over predict mortality in low-risk surgical patients^51–54^. They also consider no (ASA-ps) or few (POSSUM) surgery-related variables^55^. In 2015, the ASPRA score was implemented and tested, showing good predictive ability, as from the ROC curves, remarkably good negative predictive value for scores > 7, and a trend towards a positive association between higher scores and more severe complications as defined by the Clavien-Dindo score.

ANNs are increasingly being included in several clinical processes of shared decision-making for their capacity to detect patterns and relationships among a broad range of different input data, thus enabling decision-making under uncertain conditions^56^. ANN is a ML method evolved from the idea of simulating the human brain.

The new and easy-to-use ANN-based SUMPOT was implemented to generalize the hidden relationship between the input (i.e. patient- and surgery-related risk factors) and the output (i.e. occurrence or not of PoCs, as defined by the Clavien-Dindo score). In the testing set, the accuracy of SUMPOT in correctly identifying complicated and uncomplicated postoperative courses was 88.9% and 90.2%, respectively. Comparison with BDT performance shows that loss of interpretability due to the adoption of an ANN model is completely justified by increase in overall predictive accuracy. Indeed, the too simple BDT model, while achieving slightly better results in relation to the most represented control group class (94.1%), was totally inadequate for predicting the less represented PoC group (22.2%). Overall accuracy is 90% for ANN and 83.3% for BDT.

Since the ANN is able to progressively learn which input factors are more closely linked to the output, grading of the presumptive strength of association between single risk factors and PoCs was not necessary.

Based on data already available in the literature, we looked for the possibility to use already known preoperative risk factors as a starting point to implement an entry set of risk factors for postoperative complications. Thus, as input entries, we used a binary transformation of the risk factors already identified (i.e. 1 if the factor was present, 0 if not). Unstable coronary syndromes were excluded from the input factors since they usually require urgent treatment and certainly require postoperative elective ICU admission, irrespective of the scores. We included a BMI ratio > 25 or < 17 among the input risk factors^57–60^. Serum albumin concentration was substituted with total plasma protein value (see “Methods” section). Due to its capability to predict poor surgical outcomes, weight loss in the preoperative period was included among the variables for risk assessment to better estimate the nutritional status and frailty of the patient (see “Methods” section). Elevated serum creatinine value was added to the input risk factors if greater than 1.5 md/dl. Omission of age and sex as predictors of postoperative complications is consistent with results from other studies which demonstrate that a comprehensive frailty assessment of patients (using the same variables included in our neural network) has higher predictive power compared to age or sex alone^61,62^. Finally, we decided not to include more complex data such as cardiopulmonary exercise testing and biomarker assays, even though they could potentially increase the accuracy of the predictive score^63^. All the characteristics described above contribute to make SUMPOT an easy to apply tool in routine anesthesia practice.

The planned surgical technique (open vs. laparoscopic vs. robot-assisted) was added to the input risk factors to consider the potential advantages of less invasive surgery, as drafted in the surgical plan. Actual occurrence of a surgical complication was evaluated retrospectively in the postoperative period. This approach is consistent with that of other surgical risk score calculators that take into account the planned surgical technique. A general limitation of this approach is that, if a different technique is chosen during surgery (e.g. during conversion from laparoscopic to open procedure), also postoperative risk changes. In the case of SUMPOT, that can be easily overcome by reassessing the risk of the patient for postoperative complications. In this regard, it is worth mentioning that in our dataset the rate of laparoscopic-to-open conversion was about 4%.

Binary assessment of presence/absence of risk factors was easy to perform and required only routine clinical and surgical data available from the patient’s medical chart and his/her medical history and clinical examinations.

In the testing set, the accuracy of the model to predict the complication was defined as the rate of correct patient classifications (complicated/uncomplicated postoperative course). SUMPOT works as an input/output black-box model. Since the Clavien-Dindo score defines any deviation from normal postoperative course as “complicated”, based on treatments required during the postoperative period, we considered those patients with a Clavien score of 1 (need for morphine or extra fluids) as not complicated. This modification was made in order to add clinical meaningfulness to the prediction returned by SUMPOT. In particular, SUMPOT predicts patients with Clavien-Dindo scores > 1, thus making the score itself more clinically consistent with the chance a patient has to experience major postoperative complications and thus to benefit from a higher level of postoperative care. The accuracy of SUMPOT in predicting an uncomplicated course was 90.2%; thus, the new tool has a high likelihood of identifying patients at lower risk of developing substantial postoperative complications and who can therefore receive appropriate treatment in standard wards. The observed positive predictive value (PPV) and the negative predictive value (NPV) for SUMPOT were 61.5% and 97.9% respectively. However good these figures may appear, the performance of SUMPOT cannot be entirely described through the PPV and NPV, since the ANN technology relies on a self-training mechanism that tends to improve its predictive performance through repeated use (i.e. PPV and NPV will change over time). That is why a low initially observed PPV does not mean that the test is of lower quality. For the same reason, it is not possible to directly compare SUMPOT with more traditional surgical risk scores. Being based on ANN technology, the SUMPOT performance is better described through “accuracy” (see above). On the other hand, the new SUMPOT does not give any clues as to the severity of the expected complication, since the score itself is not graded; it simply pre-emptively classifies the surgical patient as having a complicated or uncomplicated course.

Moreover, our analysis confirms that in most cases the widely used ASA score is poorly consistent with the real clinical postoperative course (see Table 4), both for low-risk and high-risk surgical patients. Among patients with lower ASA scores (i.e., ASA $\le$ 2) postoperative complications occurred in 6.5% of cases (25 patients). Underestimating the actual risk of the patient is dangerous, not only in terms of the event “postoperative complication” but also in terms of predicting its clinical relevance. In our study, 3 patients with lower ASA scores developed substantial postoperative complications (i.e., Clavien-Dindo score 4). On the other hand, 123 patients (87.2%) with higher ASA scores (i.e., ASA > 2) did not experience postoperative complications. To consider them as “high-risk patients” for whom post-operative admission to ICU/HDU was necessary would have led to inappropriate allocation of, usually limited, hospital’s resources. These findings indicate that SUMPOT may be more efficient than ASA in identifying high-risk surgical patients and properly allocating limited ICU/HDU beds. Results of this study suggest that the ANN-based SUMPOT could support the physician in planning the most appropriate postoperative level of care.

An important advantage of SUMPOT is that preoperative evaluation of the patient is fast and simple, since it only requires clinical data easily accessible from the patient medical chart and a review of his/her medical history and clinical examinations, both aspects which are part of routine anesthesiology assessments. SUMPOT differs from other perioperative scoring systems in that it is not time-consuming and does not require recollection of complex information. This new ANN-based tool offers the advantage of automation and self-learning for progressively more accurate assessment. Our results demonstrate the efficacy of SUMPOT and underline its potential value in supporting clinical decision making.

Some limitations should be acknowledged. First, SUMPOT was developed using data from a single center. However, due to self-learning properties, it would be easy to test it in other surgical contexts. Second, the accuracy of the algorithm does not necessarily equate clinical efficacy. To be clinically meaningful, its mathematical efficacy needs to be challenged during clinical practice to test whether its use can improve the process of allocation of limited ICU/HDU resources and have an impact on postoperative outcomes. Since the ANN technology works with a self-learning black-box algorithm, even a low number of cases could be considered to validate the SUMPOT calculator. However, for further validation, a large number of cases will be included in our next analysis. Third, comparison with other scores—including the American College of Surgeons risk score calculators (the “NSQIP”) was not possible for technical reasons. In the next protocols, we will compare SUMPOT with other, more traditional surgical scores so as to consider also more severe complications and increase specificity.

## Data Availability

Custom code.
